# Soluble OX40L and JAG1 Induce Selective Proliferation of Functional Regulatory T-Cells Independent of canonical TCR signaling

**DOI:** 10.1038/srep39751

**Published:** 2017-01-03

**Authors:** Prabhakaran Kumar, Khaled Alharshawi, Palash Bhattacharya, Alejandra Marinelarena, Christine Haddad, Zuoming Sun, Shigeru Chiba, Alan L. Epstein, Bellur S. Prabhakar

**Affiliations:** 1 Department of Microbiology and Immunology, University of Illinois-College of Medicine, Chicago, IL, USA; 2 Department of Immunology, Beckman Research Institute, City of Hope, Duarte, CA, USA; 3 Department of Hematology, Faculty of Medicine, University of Tsukuba, Tsukuba, Japan; 4 Department of Pathology, University of Southern California Keck School of Medicine, Los Angeles, CA, USA

## Abstract

Regulatory T-cells (Tregs) play a pivotal role in maintaining peripheral tolerance. Increasing Treg numbers/functions has been shown to ameliorate autoimmune diseases. However, common Treg expansion approaches use T-Cell Receptor (TCR)-mediated stimulation which also causes proliferation of effector T-cells (Teff). To overcome this limitation, purified patient-specific Tregs are expanded *ex vivo* and transfused. Although promising, this approach is not suitable for routine clinical use. Therefore, an alternative approach to selectively expand functional Tregs *in vivo* is highly desired. We report a novel TCR-independent strategy for the selective proliferation of Foxp3+Tregs (without Teff proliferation), by co-culturing CD4+ T-cells with OX40 L+Jagged(JAG)-1+ bone marrow-derived DCs differentiated with GM-CSF or treating them with soluble OX40 L and JAG1 in the presence of exogenous IL-2. Tregs expanded using soluble OX40 L and JAG1 were of suppressive phenotype and delayed the onset of diabetes in NOD mice. Ligation of OX40 L and JAG1 with their cognate-receptors OX40 and Notch3, preferentially expressed on Tregs but not on Teff cells, was required for selective Treg proliferation. Soluble OX40L-JAG1-induced NF-κB activation as well as IL-2-induced STAT5 activation were essential for the proliferation of Tregs with sustained Foxp3 expression. Altogether, these findings demonstrate the utility of soluble OX40 L and JAG1 to induce TCR-independent Treg proliferation.

Regulatory T-cells (Treg) area specialized subset of T-cells which play pivotal role in suppressing self-reactive effector T-cells (Teff) and thereby help maintain the critical balance between self-tolerance and autoimmunity^1^. Depletion of Foxp3^+^ Tregs in mice leads to multi-organ autoimmunity^2^,^3^. Similarly, patients with IPEX (Immunodysregulation, Polyendocrinopathy, Enteropathy, X-linked syndrome) characterized by mutations in *FOXP3* gene suffer from multiple autoimmune diseases^4^,^5^. Restoration of functional Treg cell numbers can aid in the recovery from various experimental autoimmune diseases such as experimental encephalomyelitis^6^ and type-1 diabetes (T1D)^7^. However, translating these experimental Treg therapies into clinical practice has been challenging. Current Treg expansion protocols involve the use of anti-CD3/CD28 which can also cause concomitant expansion of Tconv/Teff cells thus limiting its utility for *in vivo* application^8^,^9^. To circumvent this limitation, highly purified Tregs are expanded *ex vivo* and then infused back into the patients. This process is cumbersome and requires good manufacturing practice (GMP) facility. Such an approach is also not suitable for routine clinical use. Moreover, *ex vivo* expansion of Tregs by repeated TCR stimulation can lead to CpG methylation within the *Foxp3* gene locus resulting in loss of Foxp3 expression^10^,^11^. Moreover, it is likely that upon adoptive transfer the *ex vivo* expanded Tregs might not only lose their Foxp3 expression, but may morph into a labile/plastic phenotype that produce pro-inflammatory cytokines^12^. Therefore, an alternative approach that can cause selective proliferation of functional Tregs, and not Teff cells, with sustained Foxp3 expression is highly desired.

Tregs differ from Tconv cells in several aspects including their activation, proliferation and function. During the steady state, upon maturation in the thymus, Tregs with self-antigen specific TCRs are positively selected and migrate to the periphery^13^,^14^,^15^. In the periphery they undergo proliferation upon interaction with dendritic cells (DCs) through their TCR^16^,^17^ while receiving survival signal from IL-2^18^,^19^. Tregs constitutively express genes such as *Ctla-4, Cd39* and *Helios*, which are associated with suppressive functions^20^. Furthermore, Tregs can suppress Teff function irrespective of their antigen specificity as well^21^,^22^. Therefore, Tregs expanded by TCR-independent methods can still retain their suppressive functions and will have significant clinical utility.

We and others have previously shown that Foxp3^+^ Treg proliferation, independent of TCR stimulation, can be induced by co-culturing them with a particular subset of dendritic cells (DCs)^23^,^24^,^25^,^26^,^27^,^28^. Bone marrow (BM) precursor cells differentiated in the presence of GM-CSF (G-BMDCs), upon co-culture with CD4^+^ T-cells, caused Treg proliferation mediated through OX40L-JAG1 co-signaling^23^. In the present study, we demonstrate that soluble OX40 L and JAG1 along with IL-2 were sufficient to cause Treg proliferation in the absence of TCR stimulation. Unlike TCR-stimulation approach, OX40L-JAG1 caused selective proliferation of Tregs with little or no proliferation of Teff cells. OX40L-JAG1 expanded Tregs were of a stable-suppressive phenotype and functionally competent. Furthermore, treatment of NOD mice with soluble OX40 L and JAG1 increased Tregs and delayed the onset of hyperglycemia in NOD mice. Using OX40 and Notch3 deficient mice, we showed that this TCR-independent Treg expansion was critically dependent on the signaling mediated through these cognate receptors for OX40 L and JAG1 respectively. Signaling studies revealed that NF-κB activation induced by OX40L-OX40 and JAG1-Notch3 interactions, and STAT5 induced by IL-2, were essential for Treg proliferation with sustained Foxp3 expression. Thus, we report a novel “TCR-independent” strategy for the selective expansion of functional Tregs which could have therapeutic implications in various autoimmune diseases including T1D.

## Results

### G-BMDCs-induced Treg proliferation is mediated through OX40L-JAG1 co-signaling in NOD mice and soluble OX40L-JAG1 are sufficient to cause Treg proliferation in the presence of IL-2

**Figure 1:**
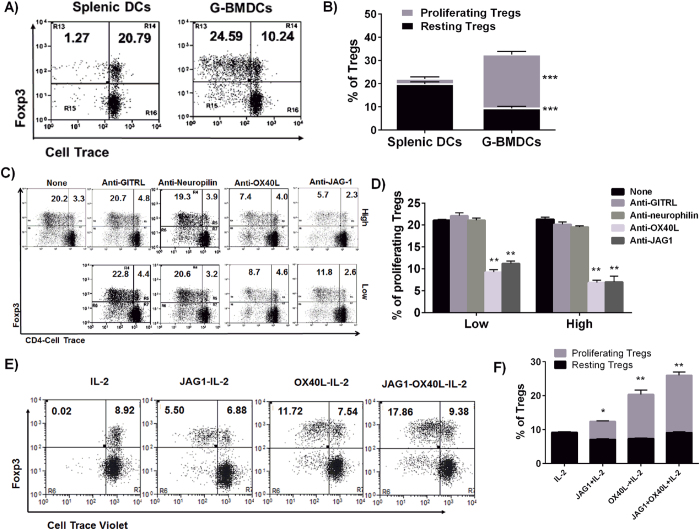
G-BMDC-induced Treg proliferation in NOD mice is mediated through OX40L-JAG1 co-signaling. (**A**) CD4+ T-cells were co-cultured with either splenic DCs or G-BMDCs in 1:1 ratio for 5 days. Extent of Treg proliferation was analyzed by flow cytometry based on cell trace violet dilution. Numbers in upper right and left quadrants indicate percentages of resting and proliferating Treg cells. (**B**) Bar graph showing percentages of resting (Black) and proliferating (Grey) Tregs. Values are expressed as Mean ± SEM (n = 3; ***p < 0.001 Vs splenic DCs). (**C**) G-BMDCs were pretreated with indicated blocking antibodies (High = 10 μgml, Low = 5 μg/ml) for 2 hrs, co-cultured with CD4+ T-cells and extent of Treg cell proliferation was measured by flow cytometry as mentioned for Fig. 1A. (**D**) Bar graph showing effect of blocking antibodies on G-BMDC induced Treg proliferation. Values are expressed as Mean ± SEM (n = 3; **p < 0.01 Vs None).(**E**) CD4+ T-cells were treated with combinations of soluble OX40 L (5 μg/ml), JAG1 (5 μg/ml) and IL-2 (10IU/ml) for 3 days as indicated. Treg proliferation was assayed by flow cytometry based on cell trace violet dilution. (**F**) Bar graph showing percentages of resting and proliferating Tregs from three independent experiments. Values are expressed as Mean ± SEM (n = 3; *p < 0.05, **p < 0.01 Vs IL-2).

**Figure 2:**
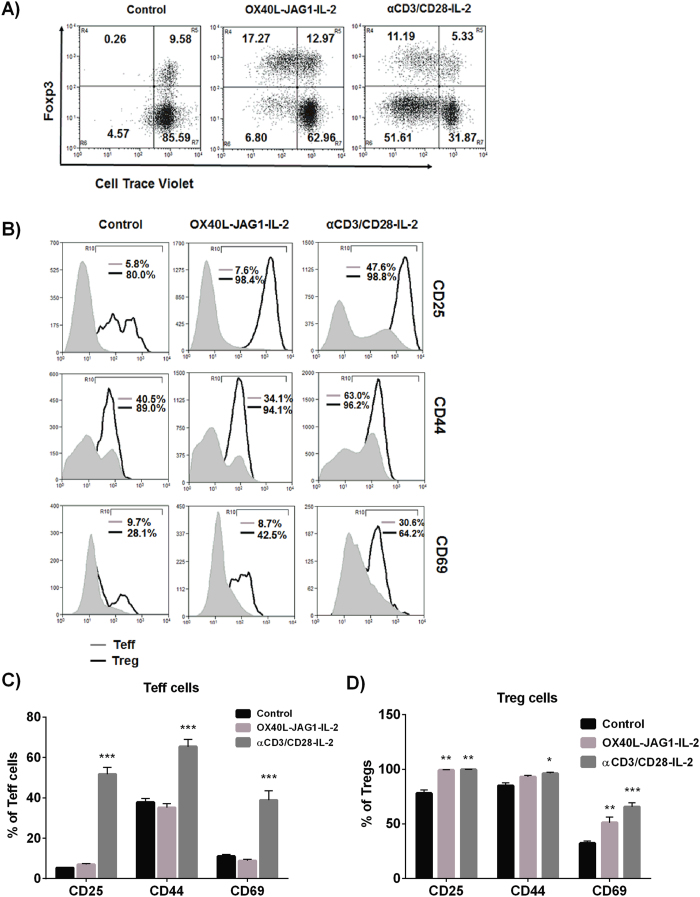
Soluble OX40L-JAG1 can cause selective Treg proliferation independent of TCR stimulation. (**A**) CD4+ T-cells were treated with IL-2 (Control), OX40L-JAG1-IL-2 and anti-CD3/CD28-IL-2 for 3 days. Extent of CD4+Foxp3- (Teff) and CD4+Foxp3+ (Treg) cell proliferation was analyzed by flow cytometry. (**B**) From the above experiments, percentages of CD25, CD44 and CD69 expressing Teff (Grey) and Treg (Black) cells were gated and indicated as numerical. (**C**,**D**) Bar graph showing percentages of Teff cells and Treg cells expressing CD25, CD44 and CD69. Values are expressed as Mean ± SEM (n = 3; *p < 0.05, **p < 0.01, ***p < 0.001 Vs control).

Upon co-culturing total CD4^+^ T-cells with splenic (SpDCs) and G-BMDCs from NOD mice for 5 days, we observed a significant (***p < 0.001) increase in selective Treg proliferation in G-BMDC co-cultures compared to co-cultures with SpDCs (Fig. 1A,B). To determine whether OX40 L and JAG1 co-signaling is involved in this G-BMDCs induced Treg proliferation, we pre-treated G-BMDCs with blocking antibodies against OX40 L, JAG1, neurolpilin and ligand for glucocorticoid-induced TNFR family related protein (GITRL) and then co-cultured with total CD4^+^ T-cells. We observed a significant reduction in Treg proliferation in the presence of blocking antibodies to OX40 L (**p < 0.01) and JAG1 (**p < 0.01) but not GITRL or neuropilin. This underscored the specific involvement of OX40 L and JAG1 signaling in G-BMDCs-induced Treg proliferation from NOD mice (Fig. 1C,D). To confirm the involvement of Notch signaling induced by JAG1, we pre-treated CD4^+^ T-cells with γ-secretase inhibitor (GSI) to inhibit Notch signaling and co-cultured with G-BMDCs. As shown in Fig. S1A, we found a dose-dependent inhibition of Treg proliferation indicating the critical role of Notch signaling. Among the various Notch receptors, Notch3 is preferentially over-expressed on Tregs when compared to Teff cells^29^. Therefore, we sorted for Notch3^−^OX40^−^, Notch3^+^OX40 L^−^, Notch3^−^OX40^+^, Notch3^+^OX40^+^ subsets of CD4^+^CD25^+^ Tregs from NOD mice and co-cultured them with G-BMDCs. The G-BMDC-induced proliferation was maximal in Notch3^+^OX40^+^ Tregs compared to Notch3+OX40- and Notch3-OX40+ Treg subsets (Fig. S1B). Next, we checked whether soluble OX40 L and JAG1 were sufficient to cause proliferation of Tregs. We treated CD4+ T-cells with soluble OX40 L and JAG1 in the presence of IL-2 without any exogenous antigenic stimulation for 3 days. Since we anticipated OX40L-JAG1 treatment not to cause Teff cell activation, exogenous IL-2 was added to maintain Treg survival in *ex vivo* cultures. As shown in Fig. 2C,D, among the different combinations tested OX40L-JAG1-IL-2 treatment caused maximum increase in the percentage of proliferating Tregs (**p < 0.01) followed by OX40L-IL-2 and JAG1-IL-2. Further, CD4+ T-cells treated with IL-2 alone or OX40L-JAG1-IL-2 were stained for proliferation marker Ki67 and percentage of Ki67+ Tregs were found to be more in OX40L-JAG1-IL-2 treated cells compared to IL-2-treated controls (Fig. S2). Taken together, these results showed that soluble OX40 L and JAG1 were sufficient to cause Treg proliferation independent of TCR stimulation in an IL-2 dependent manner.

### Soluble OX40L- JAG1-IL-2 can cause selective proliferation of Tregs independent of TCR stimulation

To validate whether OX40L-JAG1-induced Treg proliferation differs from TCR-stimulation approach, we compared the T-cell proliferation induced by TCR-dependent anti-CD3-CD28 *versus* TCR-independent OX40L-JAG1 stimulation. As shown in Fig. 2A, we observed robust proliferation of Tregs upon both OX40L-JAG1 and anti-CD3/CD28 treatment. However, unlike anti-CD3/CD28 treatment which also induced very strong Teff cell proliferation, OX40L-JAG1 treatment induced selective proliferation of Tregs without significant Teff proliferation. Analyses of activation markers expression showed a significant (***p < 0.001) increase in the percentage of Teff cells expressing CD25, CD44 and CD69 upon treatment with anti-CD3/CD28 compared to control cells (Fig. 2B–D). However, no significant difference was observed between the control and OX40L-JAG1 treated Teff cells. Moreover, Tregs from both OX40L-JAG1 and anti-CD3/CD28 treated cells had increased CD25, CD44 and CD69 expressing cells compared to control cells. These results suggested that soluble OX40L-JAG1 can cause selective proliferation of Tregs, without significantly affecting Teff cell activation and proliferation.

### Soluble OX40L-JAG1 treatment selectively induces Treg proliferation in vivo

**Figure 3:**
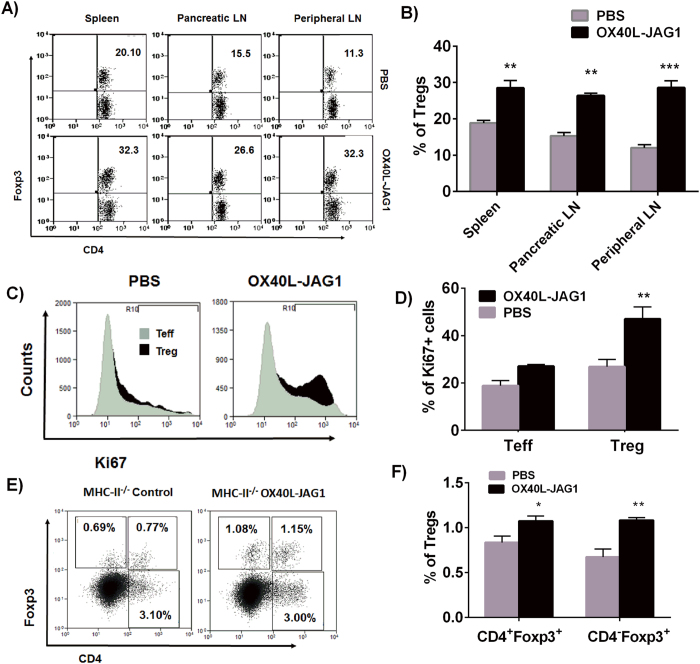
Soluble OX40L-JAG1 can cause Treg proliferation *in vivo*. (**A**) Ten week old NOD mice were treated with either PBS or 100 μg of OX40 L and JAG1 once a week for three weeks. Two weeks later, mice were sacrificed and Treg cell numbers in spleen, pancreatic LNs and peripheral LNs were analyzed. Upper and lower panels show percentages of Tregs in PBS and OX40L-JAG1 treated mice respectively. Numerical in upper right quadrant indicates percentages of Foxp3 Tregs. (**B**) Bar graph showing percentages of Treg in spleen, pancreatic and peripheral lymph nodes of PBS (Grey) and OX40L-JAG1 (Black) treated mice. Values are expressed as Mean ± SEM (n = 3; **p < 0.01, ***p < 0.001 Vs PBS). (**C**) Splenocytes from PBS and OX40L-JAG1 treated mice were stained for CD4, Foxp3 and Ki67 (proliferation marker). Ki67+ cells among CD4+Foxp3- (Grey-Teff) and CD4+Foxp3+ (Black-Treg) cells were gated and shown as histograms. (**D**) Bar graph summarizing results shown in Fig. 3C. Values are expressed as Mean ± SEM (n = 3; **p < 0.01 Vs PBS). (**E**) MHC Class-II^−/−^ mice were treated with either PBS or 100 μg of OX40 L and JAG1 as mentioned above and Treg numbers in spleens were analyzed. Numbers in the quadrants indicate percentages of CD4-Foxp3+, CD4+ Foxp3+ and CD4+Foxp3-T-cells. (**F**) Bar graph showing percentages of above mentioned cell types in spleens of PBS (Grey) and OX40L-JAG1 (Black) treated mice. Values are expressed as Mean ± SEM (n = 3; *p < 0.05, **p < 0.01 Vs PBS).

To examine whether soluble OX40L-JAG1 can cause *in vivo* proliferation of Tregs, we treated 10 week-old pre-diabetic NOD mice with soluble OX40 L and JAG1 for 3 weeks and analyzed Treg numbers in their spleen, pancreatic and peripheral lymph nodes (LN). We did not treat these mice with exogenous IL-2 as we expected IL-2 required for Treg survival to be available *in vivo*. As shown in Fig. 3A,B, we found significantly increased percentages of Tregs in the spleen (**p < 0.01), pancreatic LNs (**p < 0.01) and peripheral LNs (***p < 0.001) of OX40L-JAG1 treated mice compared to PBS treated control mice. Furthermore, to demonstrate OX40L-JAG1 induced Treg proliferation *in vivo*, we analyzed Ki67 expression in CD4 + Foxp3- (Teff), and CD4 + Foxp3+ (Treg) cells from PBS and OX40L-JAG1 treated mice. As shown in Fig. 3C,D, OX40L-JAG1 treated mice had significantly increased percentage of Ki67+ cells only in Tregs (**p < 0.01) but not in Teff population when compared to PBS treated mice. These results showed that OX40 L and JAG1 can induce preferential proliferation of Tregs both *ex vivo* and *in vivo*. Further, we treated MHC class-II deficient mice with soluble OX40L-JAG1 to determine whether these ligands can increase Tregs *in vivo* in the absence of canonical antigen presentation through MHC class-II. These mice also had reduced CD4+ T-cells and increased number of CD8+Foxp3+ T-cells when compared to wild type mice (data not shown). OX40L-JAG1 treatment significantly increased CD4 + Foxp3 + Tregs (**p < 0.05) and CD4-Foxp3+ T-cells (**p < 0.01) in these mice compared to PBS treated control mice (Fig. 3E,F). These results indicated that soluble OX40L-JAG1 can induce selective proliferation of Tregs *in vivo* independent of canonical antigen presentation to TCR.

### OX40L-JAG1-IL-2 expanded Tregs retain stable-suppressive phenotype and delay the onset of diabetes in NOD mice

**Figure 4:**
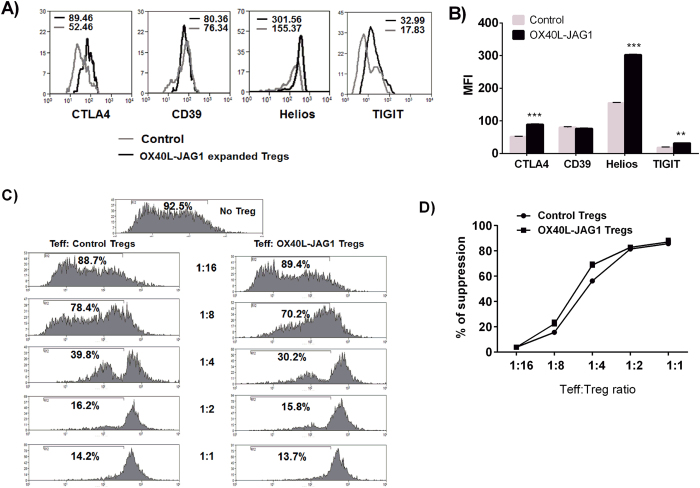
Phenotypic characterization of OX40L-JAG1-IL-2 expanded Treg cells and *in vitro* suppression assay. (**A**) Control (grey) and OX40L-JAG1 (black) expanded Treg cells were analyzed for the expression of Treg suppressive markers such as CTLA4, CD39, Helios and TIGIT and CD25 by FACS analysis. Numerical indicate respective MFI values of CTLA4, CD39, Helios and TIGIT expression in control Vs OX40L-JAG1 expanded Treg cells (n = 3). (**B**) Bar graph summarizing results showin Fig. 7A (Values represent Mean ± SEM, n = 3, **p < 0.01, ***p < 0.001 Vs Control) (**C**) Control and OX40L-JAG1 expanded CD4+CD25+ Treg cells from NOD mice were co-cultured with cell trace violet labeled fresh CD4+CD25- Teff cells at indicated ratios and stimulated with anti-CD3/CD28 for 3 days. Extent of Teff proliferation was measured by flow cytometry. (**D**) Percentage of suppression was calculated as ratio between proliferating Teff cells from Treg:Teff co-cultures to no Treg control. Graph summarizing % of suppression calculated from 4 C (Values represent Mean ± SEM, n = 4).

Next we examined whether these OX40L-JAG1 expanded Tregs retained their suppressive phenotype and functions. We analyzed the expression of suppressive markers such as CTLA4, CD39, Helios and TIGIT in Tregs from control and OX40L-JAG1 treated mice. As shown in Fig. 4A,B, OX40L-JAG1 expanded Tregs had significantly increased expression of suppressive markers such as CTLA4 (***p < 0.001), Helios (***p < 0.001) and TIGIT (**p < 0.01) when compared to control Tregs. CD39 expression was not significantly different between control and OX40L-JAG1 expanded Tregs. Furthermore, we setup an *ex vivo* suppression assay using control Tregs and OX40L-JAG1 expanded Tregs to confirm the functional competency of OX40L-JAG1-IL-2 expanded Tregs. In line with the phenotypic results, we found OX40L-JAG1 expanded Tregs to efficiently suppress Teff proliferation similar to control Tregs (Fig. 4C,D). Taken together, these results suggested that OX40L-JAG1 could expand functional Tregs without loss of their suppressive phenotype and function.

**Figure 5:**
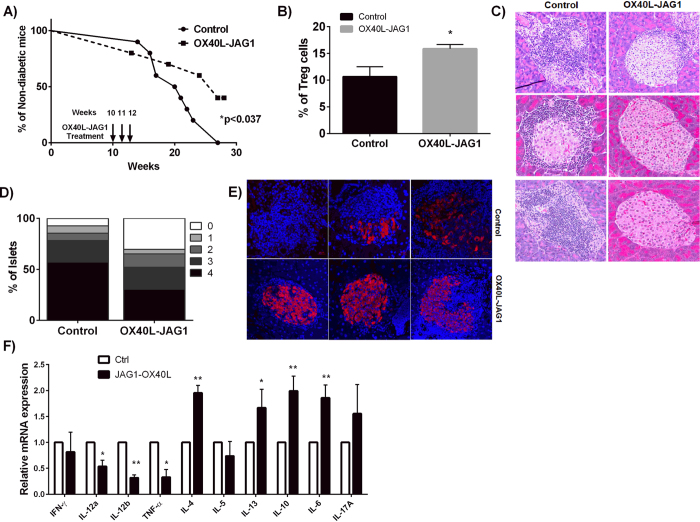
Treatment of NOD mice with OX40L-JAG1 delays onset of diabetes. (**A**) NOD mice were administered with OX40 L and JAG1 once a week for 10–12 weeks. After OX40L-JAG1 treatment blood glucose level was monitored weekly. Kaplan-Meier survival graph shows significantly delayed onset of diabetes in NOD mice upon OX40L-JAG1 treatment (*p < 0.05 Vs PBS-treated). (**B**) Spleens of 28 week old PBS and OX40-JAG1 treated NOD were analyzed for the percentage of CD4+ Foxp3+ Treg cells by flow cytometry. (**B**) Bar graph summarizing % of Tregs in spleens of PBS and OX40L-Jag1 treated mice after 28 weeks. Values are expressed as Mean ± SEM (n = 10, *p < 0.05). (**C**) H & E staining analysis of pancreatic sections from PBS and OX40L-JAG1 treated NOD mice (n = 10).(**D**) Insulitis scoring was done as described in materials & methods with the following scoring scheme: 0-no insulitis, 1-peri-islet insulitis, 2-intermediate insulitis, 3-intraislet insulitis, 4-complete islet insulitis. (**E**) Pancreatic sections were stained for insulin by immunohistochemistry (n = 10). (**F**) Splenocytes from PBS and OX40L-JAG1 treated mice were stimulated with PMA/Ionomycin and mRNA expression of indicated cytokines was analyzed by RT-qPCR. Expression values are expressed as fold induction over stimulated control cells after normalization with GAPDH. (Values represent Mean ± SEM, n = 7, *p < 0.05, **p < 0.01).

Next, we treated NOD mice with soluble OX40 L and JAG1 once a week at 10–12 weeks of age and monitored their blood glucose levels. As shown in Fig. 5A, by 27^th^ week 100% of control mice became hyperglycemic, while 40% of OX40 L and JAG1 treated mice were still normoglycemic (*p < 0.05). Additionally, we found significantly higher percentages of Tregs in the spleen of OX40 L and JAG1 treated mice (15.87 ± 0.80) relative to controls (10.67 ± 1.83; *p < 0.05, n = 10) (Fig. 5B). Examination of the pancreatic sections showed that OX40 L and JAG1 treated mice had more number of intact islets and reduced incidence of peri-insulitis (Fig. 5C). Nearly 70% of the islets from control mice showed severe insulitis with only 7.14% exhibiting normal architecture. In contrast, only 30% of the islets from OX40 L and JAG1 treated mice showed heavy infiltration and over 30% of the islets exhibited normal architecture (Fig. 5D). OX40 L and JAG1 treated mice also had higher proportion of insulin secreting islets relative to control mice (Fig. 5E). Further, we stimulated splenocytes from control and OX40L-JAG1 treated mice with PMA-Ionomycin, and analyzed their cytokine expression profile by RT-qPCR. We found reduced expression of Th1 cytokines such as IFN-γ, IL-12α (*p < 0.05), IL-12β (**p < 0.01) and TNF-α (*p < 0.05), and increased expression of Th2 cytokines such as IL-4 (**p < 0.01) and IL-13 (*p < 0.05) in the splenocytes from OX40L-JAG1 treated mice relative to controls (Fig. 5F) upon stimulation. We also noted increased expression of anti-inflammatory cytokine IL-10 (**p < 0.01) and pro-inflammatory cytokine IL-6 (**p < 0.01) in splenocytes from OX40 L and JAG1 treated mice. A recent study has shown that transgenic expression of Notch1 intracellular domain in Treg cells can cause lymphoproliferation, exacerbated Th1 responses and autoimmunity^30^. To see if a similar phenomenon was occurring, we stimulated splenocytes from control and OX40L-JAG1 treated mice with PMA-Ionomycin and stained both Treg and Teff cells for IFN-γ expression. Our results clearly showed that there was no change in the percentage of IFN-γ expressing Teff cells between control and OX40L-JAG1 treated mice, and there was barely any IFN-γ expressing Tregs in both control and OX40L-JAG1 treated mice (Fig. S3).

### Soluble OX40L-JAG1-IL-2 induced Treg proliferation is mediated through activation of OX40, Notch and IL-2R mediated NF-κB and STAT5 signaling pathways

**Figure 6:**
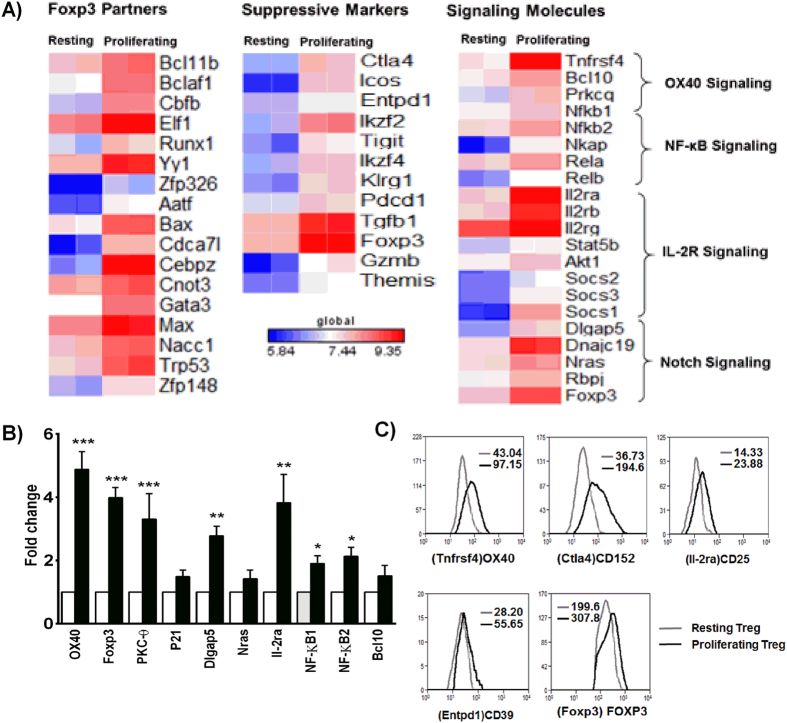
Differential gene expression analysis of resting and proliferating Treg cells. (**A**) Resting and proliferating Treg cells were sorted out based on Cell Trace violet dilution and subjected to microarray analysis to probe the differential gene expression pattern between these cells. Respective heat maps show the significantly altered expression of surface molecules, Treg functional markers, functional partners of Foxp3 and signaling molecules. (**B**) Validation of micro array results in a candidate gene approach by RT-qPCR. Fold induction values over resting Treg cells were expressed as Mean ± SEM (n = 3, *p < 0.05, **p < 0.01, ***p < 0.001 Vs Resting). (**C**) Resting and proliferating Treg cells stained for OX40, CTLA4 (CD152), CD25, CD39 and Foxp3. Respective MFI values of resting (Grey) and proliferating (black) Treg cells are indicated.

Subsequently, we analyzed for differential mRNA expression between resting and proliferating Treg cells to gain insights into the signaling pathways activated in proliferating Tregs. As shown in Fig. 6A, proliferating Tregs showed higher levels of expression of Treg functional markers such as *Ctla4, Ikzf2* (Helios), *Ikzf4* (Eos), *Tigit, Entpd1* (CD39), *Foxp3, Gzmb, Icos, Tgfb1* and *themis*; and functional partners of Foxp3 such as *Bcl11b, Bclaf1, Cbfb, Runx1, Yy1, Bax, Cebpz, Cnot3, Gata3, Max, Nacc1* and *Trp53*. Additionally, we noted increased expression of genes associated with OX40 *Tnfrsf4* (OX40), *Bcl10, Prkcq* (PKC-θ); Notch (*Dlgap, Dnajc19, Foxp3, Rbpi*); and IL-2R mediated signaling (*Il-2ra, Il-2rb, Il-2rg* and *Stat5b*) in proliferating Tregs, indicating the importance of signaling through these receptors. Furthermore, we also observed increased expression of NF-κB related genes such as *NF-κB1, NF-κB2, RelA* and *RelB* in proliferating Tregs. We validated our micro-array results in a candidate gene approach by RT-qPCR analysis and observed significantly elevated expressions of *OX40, Prkcq, Foxp3, Dlgap, Il-2ra* (*Cd25*), *NF-κB1 and NF-κB2* in proliferating Tregs (Fig. 6B). Besides, we analyzed the mean fluorescence intensities of CTLA4, CD39, OX40, CD25 and Foxp3 expression between resting and proliferating Tregs by flow cytometry. As shown in Fig. 6C, consistent with our microarray and RT-qPCR results, proliferating Tregs had increased expression of all these suppressive markers when compared with resting Tregs.

**Figure 7:**
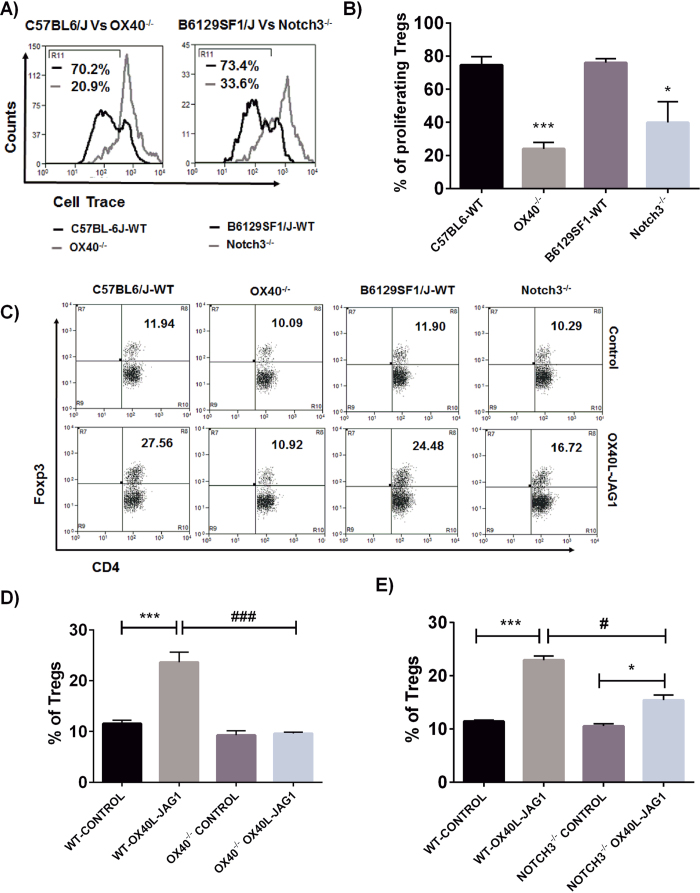
Characterization of OX40L-JAG1 induced Treg proliferation in OX40^−/−^ and Notch3−/− mice. (**A**) Extent of Treg proliferating induced by OX40L-JAG1-IL-2 was compared between C57BL6 wild type (black) Vs OX40−/− mice (grey), and B6129SF1 wild type (black) Vs Notch3−/− (grey) mice. Numerical represent percentages of proliferating Treg cells. (**B**) Bar graph summarizing results shown in (**A**). Values are expressed as Mean ± SEM (n = 3; *p < 0.05, ***p < 0.001 Vs respective wild type controls). (**C**) C57BL6 wild type, OX40^−/−^, B6129SF1 wild type and Notch3^−/−^ mice were treated with soluble OX40 L and JAG1 as mentioned in Fig. 3A. Spleens were analyzed for Treg cell numbers. Upper and lower panels show percentages of Tregs in PBS and OX40L-JAG1 treated mice. Numbers in upper right quadrant indicate percentages of Foxp3 Tregs (n = 3). (**D**,**E**) Bar graphs (**D**,**E**) show percentages of Tregs in C57BL6-WT Vs OX40^−/−^ and B6129SF1-WT Vs Notch3^−/−^ mice treated with either PBS control or OX40L-JAG1 (*p < 0.05, ***p < 0.001 Vs WT-control; ^#^p < 0.05, ^###^p < 0.001 Vs OX40^−/−^ or Notch3^−/−^ OX40L-JAG1).

OX40 L has been shown to bind to its only known cognate receptor OX40, constitutively expressed on Tregs^31^. However, JAG1 can bind to multiple receptors such as Notch1, Notch2^32^ and Notch3^33^ of which Notch3 is preferentially up-regulated in Tregs^29^,^34^. Additionally, JAG1 has been characterized as the most abundant and specific ligand for Notch3^33^.Therefore, we hypothesized that loss of either OX40 or Notch3 might negatively affect Treg proliferation induced by OX40 L, JAG1 and IL-2. We treated CD4^+^ T-cells isolated from OX40^−/−^, Notch3^−/−^ and respective wild type C57BL6 and B6129SF1 control mice with soluble OX40 L, JAG1 and IL-2 for 3 days. As shown in Fig. 7A,B, we noted a significantly lower percentage of proliferating Tregs from OX40^−/−^ (***p < 0.001) and Notch3^−/−^ (*p < 0.05) mice compared to their corresponding wild type controls. Further, we treated wild type, OX40^−/−^ and Notch3^−/−^ mice with soluble OX40 L and JAG1 for 3 weeks and analyzed Treg numbers in the spleen. As shown in Fig. 7C–E, we did not observe any significant difference in the total number of splenic Tregs among untreated OX40^−/−^, Notch3^−/−^ and the corresponding wild type control mice. Similarly, basal Foxp3 expression was also not significantly different among OX40^−/−^, Notch3^−/−^ and corresponding wild type control mice (Fig. S4). However, treatment with soluble OX40L-JAG1 caused a significant (***p < 0.001) increase in Treg numbers in both C57BL6/J and B6129SF1/J wild type mice, but not in OX40^−/−^ mice. In OX40L-JAG1 treated Notch3^−/−^ mice there was a significant increase of Tregs compared to PBS-treated Notch3^−/−^ mice (*p < 0.05), but the level of increase was still significantly less than wild type mice treated with OX40L-JAG1 (*p < 0.05 Vs OX40L-JAG1). These results suggested that although expression of OX40 or Notch3 is not required for the development of Tregs or Foxp3 expression in steady state, they are indispensable for optimal Treg proliferation induced by OX40 L and JAG1.

**Figure 8:**
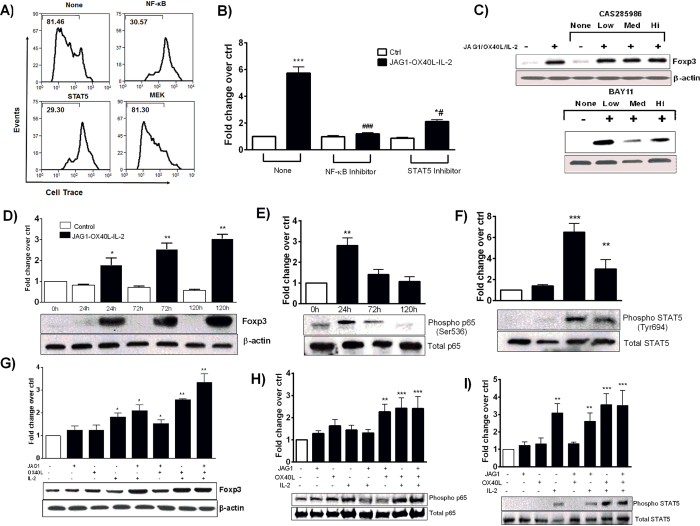
Role of NF-κB and STAT5 signaling pathways in OX40L-JAG1-IL-2 induced Treg proliferation and Foxp3 expression. (**A**) CD4+ T-cells from NOD mice were pre-treated with pharmacological inhibitors of indicated cell signaling pathways and treated with soluble OX40L-JAG1-IL-2. Effect of these pathway inhibitors on Treg cell proliferation was measured by flow cytometry analysis. (**B**) RT-qPCR analysis showing effect of inhibitors of NF-κB and STAT5 signaling pathways on Foxp3 mRNA expression (Values represent Mean ± SEM, n = 3, *p < 0.05, **p < 0.01, ***p < 0.001 Vs control, ^#^p < 0.05, ^##^p < 0.01 Vs None-OX40L-JAG1-IL-2). (**C**) Western blot analysis showing effect of inhibitors of NF-κB and STAT5 signaling pathways on Foxp3 protein expression. Western blot analysis of the time dependent effect of soluble OX40L-JAG1-IL-2 on (**D**) Foxp3 expression, (**E**) NF-κB p65 phosphorylation and (**F**) STAT5 phosphorylation in CD4+ T-cells. Western blot analysis of the effects of permutation combinations of soluble OX40 L, JAG1 and IL-2 on (**G**) Foxp3 expression, (**H**) NF-κB p65 phosphorylation, and (**I**)STAT5 phosphorylation in CD4+ T-cells (Values represent Mean ± SEM, n = 3, *p < 0.05, **p < 0.01, ***p < 0.001 Vs Control).

Since our micro array results suggested upregulation of genes associated with NF-kB and STAT5 signaling in proliferating Tregs compared to resting Tregs, we examined their role in TCR independent Treg proliferation. As shown in Fig. 8A, OX40 L, JAG1 and IL-2-induced Treg proliferation was significantly blocked by NF-κB and STAT5 inhibitors, but not by MEK inhibitor. Next, we investigated whether NF-κB and STAT5 signaling pathways were involved in the regulation of Foxp3 expression using RT-qPCR (Fig. 8B) and Western blot (Fig. 8C). While OX40 L, JAG1 and IL-2-induced Foxp3 expression was significantly down regulated in the presence of NF-κB inhibitor, whereas it was only moderately inhibited in the presence of a STAT5 inhibitor at 24 h.

Next, we carried out a time course analysis to determine the effect of OX40 L, JAG1 and IL-2 on Foxp3 expression, and NF-κBp65 and STAT5 activation. We observed a significant increase in Foxp3 expression at 24 h (*p < 0.05) which was sustained up to 120 h (Fig. 8D). While phospo-NF-κBp65 levels were maximal at 24 h (**p < 0.01) (Fig. 8E), STAT5 phosphorylation was maximum at 72 h (***p < 0.001, **p < 0.01) (Fig. 8F). Thus, it appears that Foxp3 expression was initially induced by NF-κBp65 phosphorylation and later sustained by STAT5 activation. Next, we treated CD4^+^ T-cells with different combinations of OX40 L, JAG1 and IL-2 and analyzed for Foxp3 expression, and NF-κBp65 and/or STAT5 activation. A combination of OX40 L, JAG1 and IL-2 caused maximum Foxp3 expression (**p < 0.01), followed by the combinations of OX40L-IL-2, JAG1-IL-2 and OX40L-JAG1 (Fig. 8G). Intriguingly, a significant increase in NF-κBp65 activation was observed only upon OX40 L co-treatment with JAG1 (**p < 0.01) or IL-2 or both (***p < 0.001) (Fig. 8H). Similarly, we observed impaired activation of NF-κBp65, in OX40^−/−^ CD4^+^T-cells upon OX40L-JAG1-IL-2 treatment while STAT5 activation remained unaffected (Fig. S5). Altogether, these results suggested that Treg proliferation might involve upstream signaling through OX40, Notch3 and IL-2R receptors followed by the activation of downstream NF-κB and STAT5 signaling pathways.

## Discussion

Growing body of evidence demonstrates the protective role for Foxp3+Tregs in various autoimmune diseases^6^,^7^,^35^. However, translation of Treg cell therapy to clinical settings is impeded by several limitations. One of these limitations is the inability of TCR-dependent approaches to cause selective *in vivo* expansion of Tregs^10^. Unlike stimulation with anti-CD3/CD28 which activated both Tregs and Teff cells, stimulation with OX40L-JAG1 caused selective proliferation of Tregs without activating Teffs as evidenced by no significant change in the expression of activation markers such as CD25, CD44 and CD69 on Teff cells. Differential gene expression analysis between resting vs proliferating Tregs showed up-regulation of expression of *Foxp3* and its functional partners *Gata3, Runx1, Cnot3, Cbfb, Cebpz* and *Bcl11b* in proliferating Tregs. These molecules are known to increase the functional fitness of Tregs. For example, expression of Gata3 by Tregs is essential for their migration towards the site of inflammation and to sustain Foxp3 expression under inflammatory conditions^36^. Runx and CBF-β complex have been shown to directly bind to Foxp3 promoter and increase its transcription^37^. Similarly, transcription factor Bcl11b can bind to both Foxp3 and IL-10 promoters, and regulate their expression and help confer Treg mediated protection against IBD^38^. Besides, we observed up-regulation of *Ctla-4, Helios, Tigit, Icos, Cd39, Pdcd1* and *Tgf-β1*, all of which are suppressive and stable phenotypic markers of Tregs. Further, *in vivo* treatment of NOD mice with soluble OX40 L and JAG1 resulted in a significant increase of Tregs. We also found increased expression of suppressive markers such as CTLA-4, Helios and TIGIT in Tregs expanded by *in vivo* treatment with OX40L-JAG1. CD39 expression was comparable between control and OX40L-JAG1 expanded Tregs. CTLA-4 is one of the most widely accepted mediators of Treg suppressive functions^35^. CD39, an ectonucleotidase that can hydrolyze ATP, is considered as a stability marker for Tregs and CD39^+^Foxp3^+^ Tregs have been shown to suppress both Th1 and Th17 cells^39^. TIGIT is another Treg cell specific co-inhibitory molecule. TIGIT^+^ subset of Tregs have been shown to predominantly inhibit Th1 and Th17 cells without affecting Th2 cells^40^. Helios, an Ikoras transcription factor family member, has also been reported to be associated with Treg functions^41^ and suppression of autoimmune diabetes^42^. Increased expression of these suppressive markers in OX40L-JAG1 expanded Tregs could help sustain their suppressive functions. Besides, we also confirmed the functional competency of these expanded Tregs in *ex vivo* suppressive assays.

Intriguingly, treatment of NOD mice with either OX40 L or JAG1 alone failed to significantly alter the course of diabetes (data not shown). We and others have observed that treatment of 6-week old NOD mice with either OX40 L or an anti-OX40 agonistic antibody (OX86) can increase CD4^+^CD25^+^Foxp3^+^Treg cells and protect NOD mice from developing diabetes^43^,^44^. However, treating 12-week old NOD mice with OX40 L accelerated diabetes development likely due to an increased pro-inflammatory environment associated with aging in these mice^44^. Thus, the outcome of treatment with OX40 L alone appears to be age-dependent in NOD mice. In contrast, co-treatment of 10–12-week old NOD mice with OX40 L and JAG1 significantly increased functional Treg numbers and delayed the onset of diabetes. These results indicated a critical requirement for co-signaling induced by OX40 L and JAG1 to increase and sustain functionally competent Tregs. It should be noted that we treated mice with OX40L-JAG1 once a week for only three weeks near the time of diabetes onset. However, repeated follow-up treatments might yield better results. Additionally, IL-2 deficiency in older NOD mice might have also led to poor survival of expanded Tregs^45^ and thus supplementation with IL-2 might have also increased the longevity of expanded Tregs. Interestingly, splenocytes from OX40 L and JAG1 treated mice upon PMA-Ionomycin stimulation showed reduced expression of inflammatory cytokines i.e. IFN-γ, IL-12α, IL-12β and TNF-α and increased expression of anti-inflammatory cytokines such as IL-10, IL-4 and IL-13 relative to controls. Previous reports have demonstrated the protective effects of anti-inflammatory cytokines such as IL-4, IL-10 and IL-13 in autoimmune diabetes^46^,^47^,^48^. Together, these results suggested that OX40 L and JAG1 co-treatment might have restored the balance between anti- and pro-inflammatory cytokines, and created a favorable cytokine milieu in which Tregs could proliferate and retain their suppressive functions. This notion is further evident from the reduced incidence of severe insulitis and preservation of more numbers of intact insulin secreting islets in OX40 L and JAG1 treated mice. However, further studies are required to optimize the therapeutic efficacy of this treatment.

In general, signaling required for Treg proliferation is primarily understood in the context of TCR stimulation. Previously, it has been reported that engagement of TCR with anti-CD3/CD28 can induce ZAP70, PI3K, Akt and MAPK signaling and activation of transcription factors NFAT, c-Jun, NF-κB and AP1^49^. However, the signaling that drives TCR-independent Treg proliferation is not well defined. Interestingly, while OX40L-JAG1-IL-2 induced Treg proliferation was significantly abrogated in OX40^−/−^ and Notch3^−/−^ mice, Treg proliferation induced by TCR stimulation remained unaffected in these mice (data not shown). Additionally, differential gene expression analysis revealed selective activation of genes associated with OX40, Notch and IL-2R receptor signaling in proliferating Tregs. Collectively, these results showed that the ligation of OX40 L and JAG1 with their cognate receptors OX40 and Notch3 are the major upstream events regulating this TCR-independent Treg proliferation.

The relevance of OX40 L induced signaling in Treg expansion and function has remained elusive. OX40 expression has been shown to be essential for Treg migration to inflamed sites^50^,^51^,^52^. While OX40L-OX40 stimulation can cause Treg proliferation, it could also adversely affect Foxp3 expression and Treg suppressive functions depending upon the local cytokine milieu^53^,^54^. During TCR stimulation OX40L-OX40 interaction has been shown to activate PI3K (PI-3-kinase)/PKB (protein kinase B/Akt) and NF-κB1 pathways^55^,^56^. Two members of the TRAF family of proteins such as TRAF2 and TRAF5 have been identified as key adaptor proteins recruited by OX40 to drive NF-kB1 activation^57^. In the absence of TCR stimulation, OX40 has been shown to form a signalosome containing TRAF2, CARMA1, MALT1, BCL10, PKCθ, RIP and IKKα/β/γ to cause NF-kB activation required for T-cell survival^58^. Our results showed that activation of NF-kB by OX40L-OX40 is indispensable for Treg proliferation in the absence of TCR-stimulation. OX40 deficient Tregs showed impaired NF-kB activation as well as proliferation induced by OX40L-JAG1. Several lines of evidence suggest that Notch signaling plays a positive role in Treg homeostasis by increasing Treg numbers in thymus and periphery, and by maintaining Foxp3 expression^34^,^59^,^60^. In particular, Notch3 has been shown to positively regulate nTreg development and Foxp3 expression^34^. It has been shown that Notch3 and canonical NF-κB signaling pathways could co-operatively regulate Foxp3 expression^61^. Hence, JAG1 induced Notch3 signaling, along with transactivation of NF-κB-p65 by OX40 L, could co-operatively regulate Treg proliferation and Foxp3 expression.

The role of IL-2 induced STAT5 signaling in Treg survival and stable Foxp3 expression is well established^62^. Foxp3 promoter has a STAT5 binding site^63^ through which Foxp3 expression is regulated in both human and mouse Tregs^64^. In addition, Foxp3 gene has a T-Cell Specific Demethylated Region (TSDR) in its promoter which can be demethylated through IL-2/STAT5 signaling to sustain Foxp3 expression^65^,^66^. Consistent with these earlier findings, we noted enhanced Foxp3 expression upon addition of IL-2. Thus, enhanced activation of NF-κBp65 by OX40 L and JAG1, and STAT5 signaling by IL-2 likely promoted Treg proliferation with sustained Foxp3 expression in the absence of TCR signaling. However, further studies are needed to fully understand the intricate signaling mechanisms involved in OX40L-JAG1-IL-2 induced Treg proliferation. In summary, our findings demonstrate that OX40L-JAG1 co-signaling can cause selective proliferation of Tregs in a TCR-independent mechanism which will have potential utility in treating autoimmune diseases. This TCR-independent Treg proliferation and Foxp3 expression is critically dependent on NF-kB signaling induced by OX40L-JAG1 and STAT5 signaling induced by IL-2.

## Materials and Methods

### Animals

NOD, C57BL/6 J, B6129SF1/J, OX40 and Notch3 deficient mice were purchased from Jackson Laboratories. MHC class-II deficient mice (ABBN12 (H2-Ab1)) were from Taconic biosciences. Breeding colonies were established and maintained in a pathogen-free facility of the biological resources laboratory (BRL) of the University of Illinois at Chicago (Chicago, IL). All animal experiments were approved and performed in accordance with the guidelines set forth by the Animal Care and Use Committee at University of Illinois at Chicago.

### Isolation of T-cells, and G-BMDCs-T-cells co-cultures

Spleens were excised and single cell suspensions were prepared and over 90% pure total CD4^+^ T-cells and CD4^+^CD25^+^ Tregs were isolated according to the manufacturer’s protocol (Miltenyi Biotech, CA). To derive G-BMDCs, cells isolated from femoral bones were cultured in complete RPMI-1640 containing 10% FBS supplemented with GM-CSF (20 ng/ml) for seven days. From this culture CD11c^+^ G-BMDCs were sorted and co-cultured with Cell-Trace Violet (Life technologies) labeled CD4^+^ T-cells at 1:1 ratio for five days. For TCR stimulation, cells were cultured in anti-CD3 (2 μg/ml) coated plates in the presence of anti-CD28 (2 μg/ml) (eBioscience) and IL-2 (50 IU/ml) for 72 h. In some experiments splenocytes and CD4^+^ T-cells (1 × 10^6^ /ml) were treated with recombinant mOX40 L (5 μg/ml) and mJAG1 (1 μg/ml) and mIL-2 (10 IU/ml) for 24–120 h. For proliferation experiments splenocytes and CD4^+^ T-cells were stained with Cell-Trace violet and then treated with OX40L-JAG1-IL-2.

### RNA Isolation, Micro-array and RT-qPCR analyses

Resting and proliferating Tregs were sorted based on cell trace violet dilution. Total RNA was isolated from these cells by using RNAeasy columns (Qiagen). The cDNA synthesized from total RNA was used for RT-qPCR analysis with Fast SYBR green master mix (Applied Biosystems) and gene specific primers (listed in supplementary table-1) by using AB ViiA7 RT-qPCR instrument (Applied Biosystems). Gene expression values were calculated by comparative ΔCt method after normalization to GAPDH internal control and expressed as fold change over respective controls.

Micro array analysis was performed in duplicate using the Affymetrix GeneChip Mouse Genome 430 2.0 microarray at Center for genomics core facility, University of Illinois at Chicago. Briefly, biotinylated cDNA was synthesized from total RNA using biotinylated dNTPs and allowed to hybridize with microarrays and scanned. Arrays which passed quality control tests were further subjected to gene expression analysis after normalization with housekeeping gene controls. Data were analyzed using the R-package software. Student’s t-test was used to filter differentially expressed genes Micro array has been submitted to NCBI-Gene Expression Omnibus database and publicly available (Accession No. GSE81051).

### Western blot

CD4^+^ T- cells (1 × 10^6^ cells/ml) were treated with soluble OX40 L, JAG1 and IL-2 as mentioned above. In some experiments cells were pre-treated with inhibitors of Notch (GSI-RO4929097, Selleckchem), NF-κB (BAY-11–7082, Sigma-Aldrich) and STAT5 (CAS 285986-31-4, Calbiochem) signaling for 2 h and co-treated with soluble ligands for different time intervals. Cells were washed with PBS, and lysed in Laemmli buffer (Biorad) and resolved in 10% SDS-PAGE gels. Proteins were transferred to PVDF membranes (Biorad), blocked with 5% skimmed milk and incubated with primary anti-mouse Foxp3 (1:1000, e-bioscience), anti-mouse phospho p65 (Ser536) and phospho STAT5 (Tyr694) (1:500, Cell Signaling Technology) antibodies. Blots were then washed, incubated with secondary anti-rabbit IgG-HRP and developed using ECL detection kit (Pierce Scientific). Blots were stripped and re-probed with anti-mouse β-actin-HRP antibody (1:5000; Santacruz Biotechnology), anti-mouse p65 and STAT5 (1:500, Cell Signaling Technology), and developed. Densitometry analysis was done using MyImage Analysis software (Thermo Scientific). Foxp3, pp65 and pSTAT5 signal intensities were normalized to β-actin, total p65 and STAT5 signal intensities and expressed as fold change over respective controls.

### FACS Analysis

For flow cytometry analysis cells were washed with PBS containing 0.5% BSA, either surface stained with anti-CD4-eFluor-780, CD4-FITC (eBioscience), anti-CD25-PE, anti-CTLA4-PE, anti-CD39-PE, anti-Helios-PE, anti-TIGIT-PE or fixed, permeabilized, and stained with anti-, Anti-Ki67-PE, anti-Foxp3-APC and isotype controls antibodies (eBioscience) (1:100) in dark. Samples were analyzed using CyAn ADP Analyzer (Beckman and Coulter) and data analysis was done using Summit v4.3 software.

### Suppression Assay

CD4^+^CD25^+^ Tregs sorted from control and OX40L-JAG1 treated were co-cultured with CFSE-labeled CD4^+^CD25^-^ Teff cells at 1:16, 1:8, 1:4, 1:2 and 1:1 ratios and stimulated with anti-CD3/anti-CD28. Extent of proliferation was measured by cell trace violet dilution and percentage of suppression of Teff cell proliferation was calculated as described previously^29^.

### Cell stimulation and cytokine expression analysis

Splenocytes from PBS and OX40L-JAG1 treated mice were stimulated with 500X cell stimulation cocktail (eBioscience) containing PMA and ionomycin with protein transport inhibitors for 16–24 h. mRNA expression of cytokines such as IFN-γ, IL-12α, IL-12β, TNF-α, IL-4, IL-5, IL-13, IL-10, IL-6 and IL-17 was analyzed by RT-qPCR as described above using primers listed in supplementary table-1.

### Animal experiments

Six week old female NOD mice were divided into two groups each containing13 mice. Mice were injected (i.p) with recombinant OX40 L (200 μg) and JAG1 (200 μg) on weeks 10, 11 and 12. Age and sex matched control mice received PBS. All the reagents used for animal experiments were endotoxin free (<0.1 EU/ml) when tested by using Pierce endotoxin quantification kit (Thermo scientific). Blood glucose levels were monitored weekly from week 9 to 28. On week15, three mice from each group were sacrificed and analyzed for Treg cell numbers. At the end of week 28, all animals were sacrificed and tissue sections of pancreas were subjected to histopathological examination to determine lymphocyte infiltration and β-cell destruction.

### Histopathology and immunohistochemistry

Pancreatic tissues from control and OX40L-JAG1 treated NOD mice were excised and fixed in 10% formalin overnight. Tissues were processed and stained with hematoxylin and eosin. Images captured in Aperio digital image scanner were analyzed with Aperio Image-scope viewer. Insulitis was scored independently by three individuals with the following scoring scheme: 0-no insulitis, 1-peri-islet insulitis, 2-intermediate insulitis, 3-intraislet insulitis, 4 -complete islet insulitis^67^. For immunohistochemistry, sections were stained with anti-Insulin antibody (Abcam, MA), followed by TRITC-conjugated anti-guinea pig IgG Abs (T7153) and DAPI (D9542) purchased from Sigma-Aldrich (St. Louis, MO) and subjected to confocal microscopy (Zeiss Laser Scanning Microscope; LSM 710).

### Statistical analysis

Statistical analyses were performed using Prism GraphPad (V6.0). Data were expressed as Mean ± SEM of multiple experiments. Paired Student’s t-test was used to compare two groups, whereas ANOVA with multiple comparisons was used to compare more than two groups. Differences in the frequency of hyperglycemia were determined by Kaplan-Meier survival analysis using the log-rank test. A p value < 0.05 was considered as significant.

## Additional Information

**How to cite this article**: Kumar, P. *et al*. Soluble OX40L and JAG1 Induce Selective Proliferation of Functional Regulatory T-Cells Independent of canonical TCR signaling. *Sci. Rep.* **7**, 39751; doi: 10.1038/srep39751 (2017).

**Publisher's note:** Springer Nature remains neutral with regard to jurisdictional claims in published maps and institutional affiliations.

## Supplementary Material

Supplementary Information
