# Supplementary material for: Soluble OX40L and JAG1 Induce Selective Proliferation of Functional Regulatory T-Cells Independent of canonical TCR signaling
Source: Sci Rep. 2017 Jan 3;7:39751. doi: 10.1038/srep39751 (PMC5206631; doi:10.1038/srep39751)
Supplement: Supplementary Information [file srep39751-s1.doc]

**Supplementary Data: Soluble OX40L and JAG1 Induce Selective Proliferation of Functional Regulatory T-Cells Independent of canonical TCR signaling**

Prabhakaran Kumar^*^, Khaled Alharshawi^*^, Palash Bhattacharya^*^, Alejandra Marinelarena^*^, Christine Haddad^*^, Zuoming Sun^†^, Shigeru Chiba^‡^, Alan L. Epstein^§^, and Bellur S Prabhakar^*^

Running Title: TCR–independent Treg proliferation by OX40L-JAG1

**Supplementary Table-1: PCR Primers**

| **Gene** | **Forward Primer (5’ to 3’)** | **Reverse Primer (5’ to 3’)** |
| --- | --- | --- |
| Foxp3 | CGAACATGCGAGTAAACCAATG | CTTTCACCTATGCCACCCTTA |
| GAPDH | GTGGAGTCATACTGGAACATGTA | AATGGTGAAGGTCGGTGTG |
| Notch3 | AGTGCCGATCTGGTACAACTT | CACTACGGGGTTCTCACACA |
| Prkcq | TATCCAACTTTGACTGTGGGACC | CCCTTCCCTTGTTAATGTGGG |
| NF-KB1 | ATGGCAGACGATGATCCCTAC | TGTTGACAGTGGTATTTCTGGTG |
| NF-KB2 | GGCCGGAAAGACCTATCCTACT | CTACAGACACAGCGCACACT |
| Il-2ra | AACCATAGTACCCAGTTGTCGG | TCCTAAGCAACGCATATAGACCA |
| Nras | ACTGAGTACAAACTGGTGGTGG | TCGGTAAGAATCCTCTATGGTGG |
| Dlgap5 | GTGTCACGTTTTGCCAGTCG | TCTGTTTCGCTCATACACCCT |
| OX40 | TACCTACCCCAGTGGTCACAA | ACGGATGACATAGAGTATCCCTG |
| Bcl10 | ACCAACAACCTCTCTAGGTGC | CCCTCCGGGTGGGTACATGA |
| IFN-γ | ATGAACGCTACACACTGCATC | CCATCCTTTTGCCAGTTCCTC |
| IL-12α | CCCTTGCCCTCCTAAACCAC | AAGGAACCCTTAGAGTGCTTACT |
| IL-12β | TGGTTTGCCATCGTTTTGCTG | ACAGGTGAGGTTCACTGTTTCT |
| TNF-α | CCCTCACACTCAGATCATCTTCT | GCTACGACGTGGGCTACAG |
| IL-4 | GGTCTCAACCCCCAGCTAGT | GCCGATGATCTCTCTCAAGTGAT |
| IL-5 | CTCTGTTGACAAGCAATGAGACG | TCTTCAGTATGTCTAGCCCCTG |
| IL-13 | CCTGGCTCTTGCTTGCCTT | GGTCTTGTGTGATGTTGCTCA |
| IL-6 | TAGTCCTTCCTACCCCAATTTCC | TTGGTCCTTAGCCACTCCTTC |
| IL-17 | TTTAACTCCCTTGGCGCAAAA | CTTTCCCTCCGCATTGACAC |

**Figure-S1**

**
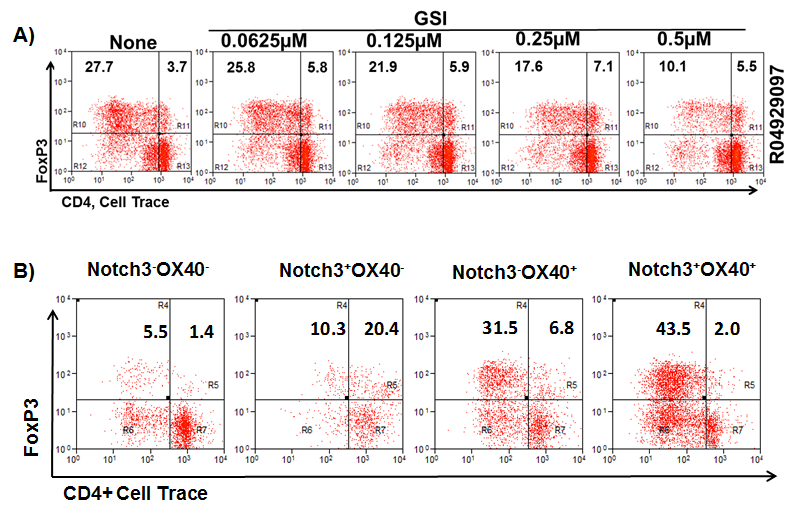
**

**Figure-S2**

**
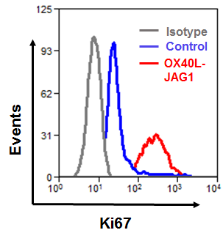
**

**Figure-S3**

**
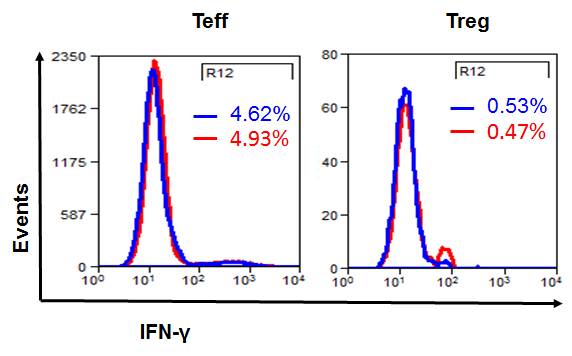
**

**
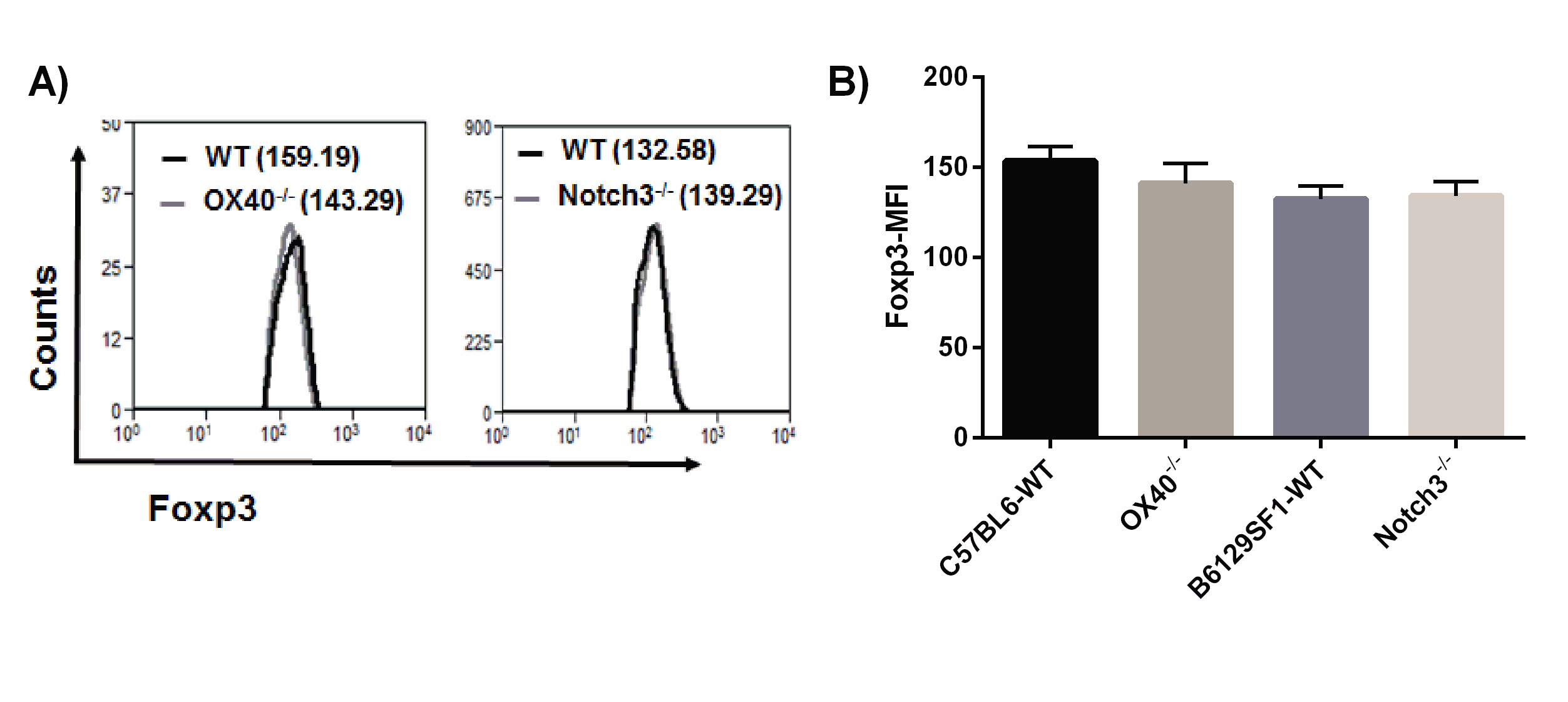
**

**Figure-S4**

**Figure-S5**

**
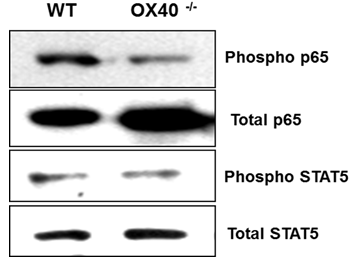
**

**Figure-S1:** A) CD4+ T-cells from NOD mice were treated with γ-secretase inhibitor (GSI)- R042929097 at indicated concentrations and then co-cultured with G-BMDCs for 5 days. Extent of proliferation was measured by flow cytometry (n=3). B) Notch3-OX40-, Notch3+OX40L-, Notch3-OX40+, Notch3+OX40+ subsets of CD4^+^CD25^+^ Treg cells sorted out and co-cultured with G-BMDCs for 5 days and extent of proliferation was analyzed (n=2).

**Figure-S2:** A) Histograms showing percentage of Ki67+ Foxp3+ Tregs in cells treated with IL-2 alone (Blue) or OX40L-Jag1-IL-2 (Red). Grey shaded curves indicate staining with isotype- matched control antibody.

**Figure-S3:** A) Histograms showing percentage of IFN-γ+ Foxp3- Teff cells and IFN-γ+Foxp3+ Treg cells in splenocytes from control (Blue) and OX40L-JAG1 (Red) stimulated with PMA-Ionomycin.

**Figure-S4:** A) Histograms showing Foxp3 MFI values between C57BL6 wild type (black) Vs OX40-/- mice (grey), and B6129SF1 wild type (black) Vs Notch3-/- (grey) mice. B) Bar graph summarizing results shown in Fig-S2A.

**Figure-S5**: CD4+ T-cells from C57BL6 and OX40^-/-^ mice were treated with OX40L, JAG1 and IL-2. Extent of NF-κB p65 and STAT5 phosphorylation was analyzed by Western blot.
